# Low ALDH1A1 expression in relation to nodal metastasis and survival in tongue squamous cell carcinoma

**DOI:** 10.1371/journal.pone.0345274

**Published:** 2026-04-10

**Authors:** Sarina Moradianlotfi, Tahere Yousefi, Afsaneh Goudarzi, Ahmad Reza Shamshiri, Samira Derakhshan

**Affiliations:** 1 School of Dentistry, Tehran University of Medical Sciences, Tehran, Iran; 2 Department of Pathology, Amir-Alam Hospital, Tehran University of Medical Sciences, Tehran, Iran; 3 Department of Clinical Biochemistry, School of Medicine, Shahid Beheshti University of Medical Sciences, Tehran, Iran; 4 Research Center for Caries Prevention, Dentistry Research Institute, Tehran University of Medical Sciences, Tehran, Iran; 5 Department of Community Oral Health, School of Dentistry, Tehran University of Medical Sciences, Tehran, Iran; 6 Department of Oral and Maxillofacial Pathology, School of Dentistry, Tehran University of Medical Sciences, Tehran, Iran; All India Institute of Medical Sciences, INDIA

## Abstract

**Background/Aims:**

Multiple biomarkers have been proposed to identify cancer stem cells in tongue squamous cell carcinoma. This study evaluated ALDH1A1, an ALDH1 subtype implicated in head and neck cancer stem cells, and examined its association with histopathological features (depth of invasion, worst pattern of invasion, perineural invasion, grade, inflammation, TNM stage) and prognostic outcomes in tongue tumors.

**Materials and methods:**

This cohort study included 55 confirmed cases of tongue squamous cell carcinoma retrieved from the pathology archives of the Cancer Institute, Imam Khomeini Hospital Complex, Tehran, Iran. Four-μm sections were stained immunohistochemically using a mouse monoclonal ALDH1A1 antibody. Histopathological variables were obtained from pathology reports and/or reassessed on slides. Patient contact information was used to follow up on recurrence and death.

**Results:**

High and low ALDH1A1 expression was observed in 27 (49.1%) and 28 (50.9%) cases, respectively, with follow-up periods ranging from 1 to 62 months. The mean age of patients was 57.24 ± 17.82 years, with a range of 24–101 years. The study included 26 men and 29 women. Low ALDH1A1 expression was linked to regional lymph node metastasis (p = 0.01). Disease-specific survival was independently associated with low ALDH1A1 expression (p = 0.045) and T category (p = 0.01). Overall survival was independently associated with age (p-value = 0.04) and T category (p-value = 0.02).

**Conclusion:**

Low ALDH1A1 expression in tongue squamous cell carcinoma was associated with regional lymph node metastasis and reduced disease-specific survival. Larger studies, including analyses across different oral subsites, are needed to clarify the relationship between ALDH1A1 expression and clinicohistopathological factors in oral squamous cell carcinoma.

## Introduction

Oral squamous cell carcinoma (OSCC) is the most common malignancy of the head and neck region worldwide [1]. The tongue is the most frequently involved oral subsite and is often associated with poorer outcomes, including higher rates of regional and distant metastasis and recurrence [2–4]. Tongue squamous cell carcinoma (TSCC) has a 5-year survival below 50% and a rising incidence [5]. Biomarkers that support earlier detection and more accurate prognostication may improve patient stratification and outcomes [6,7]*.*

One proposed explanation for the aggressive behavior of OSCC is the presence of cancer stem cells (CSCs), a tumor cell subpopulation with self-renewal capacity that contributes to tumor maintenance, metastasis, treatment resistance, intratumoral heterogeneity, and recurrence [8,9]. CSCs have been investigated using markers such as SOX2, OCT4, and CD44, but identification remains challenging because CSCs share pathways and phenotypic features with normal stem cells. Consequently, additional and more informative biomarkers are still needed. Several diagnostic and prognostic markers have been proposed in OSCC [10]. Among candidate CSC-related markers in head and neck cancer, aldehyde dehydrogenase 1 (ALDH1) has received particular attention [8,11]. ALDH1 and its isoform ALDH1A1 are expressed in adult stem cells and have also been reported as markers of CSCs in primary tumors [12]. Targeting ALDH1A1 has been suggested as a potential strategy to affect CSCs in drug-resistant, chemo-resistant, and locally advanced head and neck cancers, with limited impact on normal stem cells [12,13]. In OSCC, higher ALDH1A1 expression has been linked to advanced stage and worse prognosis in some studies, although findings across cohorts are not fully consistent [13].

Tumor behavior is also reflected in histopathological features at the tumor invasion front, often considered the most representative area for assessing invasiveness. Depth of invasion (DOI) and the pattern of invasion are increasingly used for risk stratification, although reported associations between DOI and outcomes such as overall survival and disease-free survival remain variable [10,14]. DOI and perineural invasion (PNI) are established prognostic factors, and the worst pattern of invasion (WPOI) has recently been incorporated into routine histopathological reporting in many settings [3]. DOI is generally regarded as more informative than tumor thickness and is associated with a higher risk of local recurrence and lymph node metastasis [15,16].

Given the potential links between CSC biology and invasive tumor morphology, this study aimed to assess ALDH1A1 expression in TSCC and examine its association with clinicopathological and prognostic variables, with particular emphasis on DOI and WPOI as histopathological markers of invasiveness.

## Materials and methods

### Sampling

In this mixed cohort study, 55 formalin-fixed, paraffin-embedded specimens with a definitive diagnosis of TSCC were retrieved from the pathology archives of the Cancer Institute, Imam Khomeini Hospital Complex, Tehran University of Medical Sciences, Tehran, Iran. Case selection was performed by identifying eligible cases starting from March 2021 and then moving backward consecutively to achieve an adequate follow-up period. The study was initiated on September 23, 2023, and recruitment ended on March 20, 2025.

### Ethical considerations

The Imam Khomeini Hospital Complex Ethics Committee reviewed and approved the study protocol in April 2023, with registration ID IR.TUMS.IKHC.REC.1402.056.

The informed consent was obtained verbally over the phone. The researchers explained the study process and sent a file to the patients’ contact numbers, which included the date of contact, the researchers’ names and contact information, and a statement that the patient had verbally approved to participate in the study. After sending this file, patients were contacted again to confirm their participation. All participants confirmed their cooperation during the second phone call. Also, for the first time in Iran, hospitals and schools affiliated with Tehran University of Medical Sciences have established a registry for OSCC patients (TUMS OSCC registry). Patients who joined the registry have signed a consent form. We have double-checked our data against the OSCC registry, and all of our patients are included in the TUMS OSCC registry. Since patients signed a consent form for the TUMS OSCC registry, we did not request an additional written consent.

### Eligibility criteria

The inclusion and exclusion criteria of this study are shown in Table 1.

**Table 1:** Inclusion and exclusion criteria.

Inclusion criteria	Exclusion criteria
Definite TSCC diagnosis in the pathology report.	Samples without adequate information related to our study.
Samples of the primary tumors.	Small samples with insufficient tissue to prepare microscopic slides.
Patients without a history of radiotherapy/chemotherapy.	Patients whose contact information was incorrect or could not be contacted.

### Variable assessment

Demographic information of patients was collected from archived patient files, which were accessed from September 23, 2023, till the end of the study. After recording data for variables such as gender, age, date of diagnosis, and topography, the patients were included in the study. The researchers were blinded to information that could identify individual participants during the data collection. The histopathological grade, depth of invasion (DOI), and worst pattern of invasion (WPOI) were obtained from the patients’ pathology reports. If any of these variables were not reported, the Hematoxylin and Eosin (H&E) slides of the relevant samples were re-examined by two oral and maxillofacial pathologists. In case of discrepancies, the two pathologists discussed to reach a consensus.

Histopathological tumor grading was conducted according to the World Health Organization classification of head and neck tumors; it was defined as well-differentiated or grade I, moderately differentiated or grade II, and poorly differentiated or grade III [17].

The depth of invasion (DOI) was measured in millimeters (mm). The cut-off points for DOI were set at 5 and 10 mm, according to the American Joint Committee on Cancer Eighth Edition Cancer Staging manual [15].

The worst pattern of invasion (WPOI) includes five stages: WPOI1 is characterized by a broad pushing invasive front, WPOI2 is characterized by broad pushing fingers or separate tumor islands, WPOI3 consists of invasive islands with more than 15 cells per island, WPOI4 consists of invasive islands with fewer than 15 cells per island or single-cell invasion, and WPOI5 involves tumor satellites located more than 1 mm away from the primary tumor [18].

To assess inflammation, the section with the highest level of inflammation was selected, and cell counting was performed as follows: Mild had fewer than 500 inflammatory cells, moderate had between 500 and 1,000, and severe had more than 1000 inflammatory cells [19].

TNM staging was obtained based on the AJCC Cancer Staging Manual, Eighth Edition (2017) [20].

### Immunohistochemistry staining

Four-µm-thick slices were prepared, and immunohistochemical (IHC) staining was performed using a mouse monoclonal ALDH1A1 antibody (ZM71, MonoMAb™, Zeta Corporation, Southern California, USA) according to the manufacturer’s instructions. The slides were placed in the oven for 24 hours. Three toluene containers were used for deparaffinization, with the slides placed in each container for 10 minutes. Next, four containers were utilized for hydration: slides were placed in the first container with 100% ethanol, the second with 96% ethanol, and the third with 70% ethanol for 2 minutes each. The slides were then transferred to the last container containing double-distilled water for 5 minutes. To block internal peroxidase activity, 100 µL of peroxidase-blocking reagent was applied to each slide for 10 minutes in the dark. Next, the slides were immersed three times for 5 minutes each in phosphate-buffered saline (PBS). Antigen reassortment was performed using citrate buffer (×1), pH 6, for 20 minutes at 90°C in a Bain-Marie, followed by a 20-minute cooling period to room temperature. The slides were washed with double-distilled water for 5 minutes. For primary antibody incubation, 100 µL of the antibody was applied to each slide, which was then incubated for one hour in a humid chamber at room temperature. The slides were washed three times with PBS for 5 minutes each. A 100 µL volume of primary antibody amplifier was applied, and the slides were incubated for 20 minutes. The slides were then washed three times with PBS for 5 minutes each. Next, 100 µL of secondary antibody was added, and the slides were placed in the dark for 30 minutes at room temperature. Afterward, the slides were washed three times with PBS for 5 minutes each. A microtube containing one cc of DAB substrate buffer was prepared, and a drop of DAB chromogen was added; this mixture was protected from light. Then, 100 µL of this solution was applied to each slide and incubated for 5 minutes. The slides were washed with double-distilled water and then stained with hematoxylin. The dehydration process was followed, using three containers with 96% ethanol, 100% ethanol, and toluene, each for 2 minutes. Finally, mounting was completed, and the slides were covered with microscopic lamelae.

### ALDH1A1 measurement

Immunohistochemically stained slides were scored by two pathologists. Immunoreactivity in the membrane and cytoplasm of tumor cells was deemed positive, whether patchy or diffuse. The assessment included staining intensity and the proportion of stained cells. Intensity was rated on a 0–3 scale at ×400 magnification: 0 indicates no staining, 1 indicates mild staining, 2 signifies moderate staining, and 3 represents severe staining. The proportion of tumor cells was scored as 0 for ≤5%, 1 for 6–25%, 2 for 26–49%, and 3 for ≥50%. The final score was obtained by multiplying the intensity and proportion, which ranged from 0 to 9. A cut-off of 2 was used: scores ≤2 were classified as low or negative expression, and scores >2 as high or positive expression [13].

### Patient follow-up

Patients were contacted by phone according to call guidelines [21]. At the beginning of the phone interview, patients were asked for permission to record the session. All participants agreed to this request. During the telephone interview, patients were asked about the presence or absence of recurrence and the timing of their last doctor’s visit. Patients who had more than three months since their previous visit were requested to have a visit with the pathologist. If patients did not answer, their secondary contact number was tried. In this case, we asked that the patients themselves be involved. If the patient was not available due to death, we inquired about the cause of death. If the patient was unavailable for any other reason, we scheduled another attempt to contact them. If the patient’s contact information was incorrect or they did not respond after multiple attempts—five times at different hours—they would be excluded from the study.

### Statistical analysis

Data were analyzed using SPSS version 27 (IBM SPSS Statistics for Windows, Armonk, NY, USA). Qualitative data were presented as percentages. Quantitative variables were presented as means and standard deviations, and variables not following a normal distribution were described using medians and interquartile ranges. The Mann-Whitney test was used to compare qualitative variables (inflammation, grade, DOI, WPOI, PNI, stage, and topography) and non-normally distributed quantitative variables between two groups: high and low expression. Overall survival (OS) and disease-specific survival (DSS) times were calculated from the date of the pathology report at diagnosis until the last follow-up. To assess recurrence-free survival (RFS), the period from the date of surgery as recorded in the pathology report to the last follow-up was used. Patients who died without receiving additional therapy following surgery to achieve complete remission were excluded from the RFS analysis. Cox regression analysis was performed to evaluate survival, taking into account confounding factors. A p-value of less than 0.05 was considered statistically significant.

## Results

### Patient description

Fifty-five TSCC specimens were selected from the archives of the pathology department of the Cancer Institute, Imam Khomeini Hospital Complex, Tehran University of Medical Sciences, Tehran, Iran. The anonymized dataset is shown in S1 Table. The mean age of the patients was 57.24 ± 17.82, ranging from 24 to 101 years. The study included 26 men and 29 women.

### ALDH1A1 expression in its correlation with clinicopathological features

**Fig 1:**
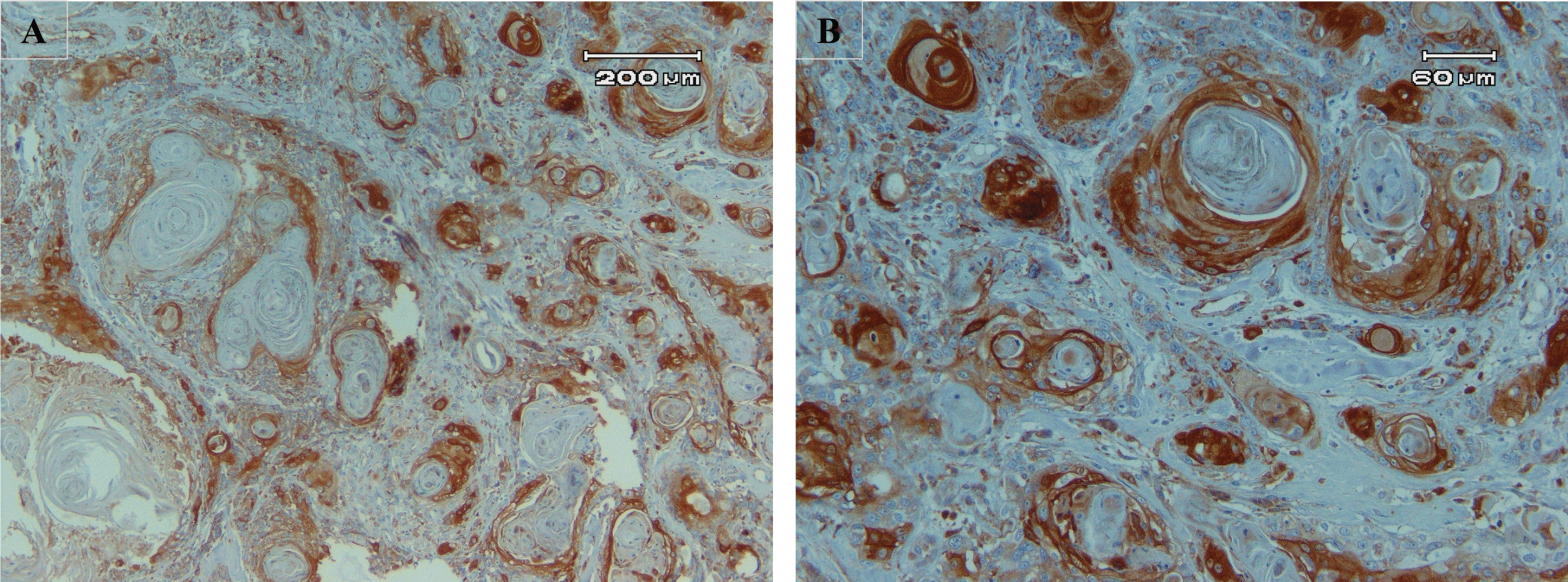
High expression of ALDH1A1. **A.** Severe staining in more than 50% of cells (x100). **B.** Closer view (x400).

**Fig 2:**
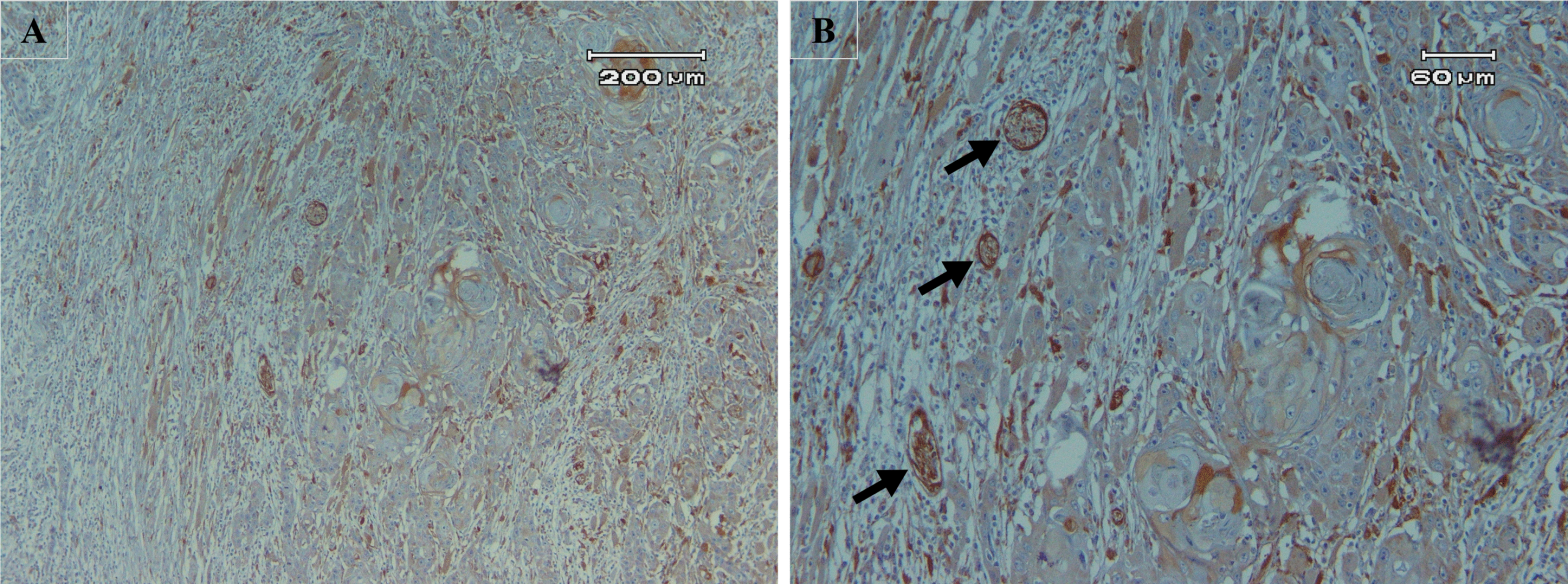
Low expression of ALDH1A1. **A.** Mild staining in less than 25% of cells (x100). **B.** Perineural invasion (black arrows, x400).

The results of the IHC assay on 55 TSCC specimens showed that 27 of 55 (49.1%) exhibited high ALDH1A1 expression (Fig 1), while 28 of 55 (50.9%) showed low ALDH1A1 expression (Fig 2). The Mann-Whitney test results are presented in Table 2. Among all variables evaluated with this test, only regional lymph node metastasis (N) was significantly associated with low ALDH1A1 expression (p-value=0.01). Twenty-nine patients were under 60 years old; 16 (55.2%) were in the high-expression group, and 13 (44.8%) were in the low-expression group. Twenty-six patients were 60 years or older, with 11 (42.3%) in the high-expression group and 15 (57.7%) in the low-expression group. Eleven women and 16 men, out of 29 and 26, respectively, were in the high-expression group. The mean depth of invasion for high and low ALDH1A1 expression groups was 8.11±3.04 and 9.39±5.62, respectively. The DOI (p-value=1.00), WPOI (p-value=0.49), PNI (p-value=0.70), grade (p-value=0.30), and stage (p-value=0.14) showed no significant association with ALDH1A1 expression.

**Table 2:** Clinicohistopathological variables. ALDH1A1 expression and its association with clinicopathological variables analysed using the Mann-Whitney test.

		Total	High expression		Low expression		p-value
			Count	%	Count	%	
**Inflammation**	**Mild**	2	1	50.0	1	50.0	0.85
	**Moderate**	15	7	46.7	8	53.3	
	**Severe**	38	19	50.0	19	50.0	
**Topography**	**Ventral and lateral borders**	22	11	50.0	11	50.0	0.91
	**Others**	33	16	48.5	17	51.5	
**Grade**	**I**	19	10	52.6	9	47.4	0.30
	**II**	23	13	56.5	10	43.5	
	**III**	13	4	30.8	9	69.2	
**DOI**	**1-4 mm**	7	3	42.9	4	57.1	1.00
	**5-9 mm**	27	14	51.9	13	48.1	
	**≥10 mm**	21	10	47.6	11	52.4	
**WPOI**	**1**	0	0	0.0	0	0.0	0.49
	**2**	3	0	0.0	3	100.0	
	**3**	11	4	36.4	7	63.6	
	**4**	28	18	64.3	10	35.7	
	**5**	13	5	38.5	8	61.5	
**PNI**	**Present**	32	15	46.9	17	53.1	0.70
	**Not present**	23	12	52.2	11	47.8	
**Stage**	**I**	6	4	66.7	2	33.3	0.14
	**II**	11	7	63.6	4	36.4	
	**III**	25	11	44.0	14	56.0	
	**IV**	13	5	38.5	8	61.5	
**T**	**1**	13	6	46.2	7	53.8	0.79
	**2**	27	15	55.6	12	44.4	
	**3**	12	4	33.3	8	66.7	
	**4**	3	2	66.7	1	33.3	
**N**	**0**	25	17	68.0	8	32.0	**0.01**
	**1**	20	7	35.0	13	65.0	
	**2**	7	2	28.6	5	71.4	
	**3**	3	1	33.3	2	66.7	
**M**	**0**	51	26	51.0	25	49.0	0.32
	**1**	4	1	25.0	3	75.0	

### ALDH1A1 expression and patient survival

Thirteen relapses and 23 deaths were recorded, with three deaths unrelated to the disease. All patients were included in the OS and DSS analyses; however, three patients who expired before treatment completion were excluded from the RFS analysis (all in the high-expression ALDH1A1 group). The last follow-up was conducted on July 20, 2024. The median follow-up duration for OS in survivors was 47 months (range, 1-62 months). The DSS and RFS mean follow-up duration in the ALDH1A1 high-expression group was 51.87 (SE=±3.67) and 51.50 (SE=±3.80), and in the ALDH1A1 low-expression group was 35.36 (SE=±4.56) and 43.88 (SE=±4.45), respectively.

### Cox regression survival analysis

The results of the simple Cox regression analyses for DSS, OS, and RFS are shown in Tables 3–5, respectively. Although RFS had no association with the variables in this study, as demonstrated by multiple Cox regression analyses, DSS was linked to lower expression of ALDH1A1 (HR=0.37, 95% CI, p-value=0.045) and a higher T category (HR=3.15, 95% CI, p-value=0.01). OS was associated with age (HR = 2.48, 95% CI, p-value = 0.04) and T category (HR = 2.70, 95% CI, p-value = 0.02) independently (Table 6).

**Table 3:** Disease-specific survival rates. Disease-specific survival rates and clinicopathological variables in tongue squamous cell carcinoma using simple Cox regression.

		n	HR	CI 95%		p-Value
				Lower	Upper	
**Age**	**<60 years**	29	2.54	1.01	6.37	0.05
	**≥60 years**	26	Ref.			
**Gender**	**Male**	26	Ref.			
	**Female**	29	0.53	0.21	1.34	0.18
**Inflammation**	**Mild/ Moderate**	17	1.41	0.51	3.90	0.50
	**Severe**	38	Ref.			
**Topography**	**Ventral and lateral borders**	22	2.08	0.75	5.72	0.16
	**Others**	33	Ref.			
**Grade**	**I**	19	0.81	0.26	2.55	0.71
	**II**	23	0.87	0.28	2.66	0.81
	**III**	13	Ref.			
**DOI**	**1-9 mm**	34	1.44	0.59	3.47	0.42
	**≥10 mm**	21	Ref.			
**WPOI**	**1-4**	42	1.10	0.40	3.05	0.84
	**5**	13	Ref.			
**PNI**	**Present**	32	Ref.			
	**Not present**	23	2.54	0.92	7.00	0.07
**Stage**	**I-II**	17	0.12	0.03	0.58	**0.01**
	**III**	25	0.42	0.17	1.08	0.07
	**IV**	13	Ref.			
**T**	**1-2**	40	3.39	1.41	8.15	**0.01**
	**3-4**	15	Ref.			
**N**	**0**	25	4.18	1.39	12.51	**0.01**
	**1-3**	30	Ref.			
**ALDH1A1**	**High expression**	27	Ref.			
	**Low expression**	28	0.35	0.13	0.90	**0.03**

**Table 4:** Overall survival rates. Results of simple Cox regression analyzing the overall survival rate and its association with clinicopathological variables in tongue squamous cell carcinoma.

		n	HR	CI 95%		p-Value
				Lower	Upper	
**Age**	**<60 years**	29	2.54	1.07	5.99	**0.03**
	**≥60 years**	26	Ref.			
**Gender**	**Male**	26	Ref.			
	**Female**	29	0.76	0.33	1.74	0.52
**Inflammation**	**Mild/ Moderate**	17	1.09	0.45	2.66	0.84
	**Severe**	38	Ref.			
**Topography**	**Ventral and lateral borders**	22	1.96	0.77	4.98	0.15
	**Others**	33	Ref.			
**Grade**	**I**	19	1.20	0.41	3.52	0.74
	**II**	23	0.86	0.28	2.64	0.80
	**III**	13	Ref.			
**DOI**	**1-9 mm**	34	1.58	0.70	3.59	0.27
	**≥10 mm**	21	Ref.			
**WPOI**	**1-4**	42	1.19	0.47	3.01	0.71
	**5**	13	Ref.			
**PNI**	**Present**	32	Ref.			
	**Not present**	23	1.89	0.77	4.60	0.16
**Stage**	**I-II**	17	0.16	0.04	0.58	**0.005**
	**III**	25	0.40	0.16	0.96	**0.04**
	**IV**	13	Ref.			
**T**	**1-2**	40	2.28	1.21	6.37	**0.02**
	**3-4**	15	Ref.			
**N**	**0**	25	3.07	1.21	7.80	**0.02**
	**1-3**	30	Ref.			
**ALDH1A1**	**High expression**	27	Ref.			
	**Low expression**	28	0.51	0.22	1.18	0.11

**Table 5:** Recurrence-free survival rate. Results of simple Cox regression analyzing recurrence-free survival rate and its association with clinicopathological variables in tongue squamous cell carcinoma.

		n	HR	CI 95%		p-Value
				Lower	Upper	
**Age**	**<60 years**	29	1.21	0.41	3.63	0.72
	**≥60 years**	23	Ref.			
**Gender**	**Male**	25	Ref.			
	**Female**	27	1.18	0.40	3.52	0.76
**Inflammation**	**Mild/ Moderate**	17	1.11	0.34	3.16	0.86
	**Severe**	35	Ref.			
**Topography**	**Ventral and lateral borders**	20	2.57	0.70	9.35	0.15
	**Others**	32	Ref.			
**Grade**	**I**	19	1.05	0.25	4.40	0.95
	**II**	20	0.99	0.24	4.15	0.99
	**III**	13	Ref.			
**DOI**	**1-9 mm**	33	1.11	0.36	3.40	0.85
	**≥10 mm**	19	Ref.			
**WPOI**	**1-4**	40	0.57	0.12	2.59	0.47
	**5**	12	Ref.			
**PNI**	**Present**	30	Ref.			
	**Not present**	22	1.90	0.58	6.19	0.28
Stage	**I-II**	17	0.51	0.13	2.06	0.35
	**III**	23	0.47	0.13	1.77	0.26
	**IV**	12	Ref.			
**T**	**1-2**	39	1.08	0.30	3.93	0.91
	**3-4**	13	Ref.			
**N**	**0**	25	1.78	0.58	5.46	0.31
	**1-3**	27	Ref.			
**ALDH1A1**	**High expression**	27	Ref.			
	**Low expression**	25	0.67	0.23	2.01	0.48

**Table 6:** Multiple Cox regression. Results of Multiple Cox regression in Disease-specific survival (DSS) and Overall survival (OS) rates.

		HR	CI 95%		p-Value
			Lower	Upper	
DSS	ALDH1A1	0.38	0.14	0.98	**0.045**
	T	3.15	1.30	7.60	**0.01**
OS	Age	2.48	1.05	5.87	**0.04**
	T	2.70	1.18	6.19	**0.02**

## Discussion

In this study, low ALDH1A1 expression assessed by immunohistochemistry in TSCC was associated with regional lymph node metastasis. In addition, disease-specific survival was independently associated with ALDH1A1 expression and T category in the TNM classification.

TSCC is the most common malignancy within the oral cavity and is characterized by a rising incidence and poor prognosis. Because biological behavior and outcomes vary across oral cavity subsites, many studies have explored subsite-specific biomarkers for diagnosis and prognostic stratification [22]. However, most proposed biomarkers in TSCC remain far from routine clinical implementation, and there is still a need for markers that can better predict patient outcomes [6].

ALDH1A1, as a prognostic biomarker, was correlated with both poor and favorable prognosis and clinical outcomes in many cancers [12]. In Leinung et al.‘s study, ALDH1A1-positive patients exhibited worse survival rates [23]. The same results were concluded by Szafarowski et al, as ALDH1A1-positive were correlated to 5-year overall survival rates [24]. In Gupta et al.’s study, high ALDH1A1 expression was associated with high mortality rates and poor prognosis in OSCC [13]. Qian et al. showed that ALDH1A1 expression serves as a predictor of poor prognosis in patients with HNSCC [25]. Xu et al. discussed the expression of ALDH1A1 as a prognostic factor in HNSCC, but concluded that it is not an independent prognostic factor for the survival of HNSCC patients [26]. Only three specimens were ALDH1A1-negative in our study and were assigned to the low-expression group, with a worse disease-specific survival rate. The detection method we used, which was IHC, could potentially affect the results.

A relation between low ALDH1A1 expression and poorer survival outcomes has been observed in some carcinomas [27–30], including OSCC [28]. In pancreatic cancer, patients with low ALDH1A1 expression experienced shorter median OS and RFS than those with high ALDH1A1 expression. Additionally, low ALDH1A1 expression served as an independent prognostic marker for OS and was the only significant prognostic factor for recurrence-free survival, as confirmed by Cox regression [29]. These findings align with another study on hepatocellular carcinoma, which concluded that patients with low mRNA and protein expression of ALDH1L1 have a poorer prognosis, indicating that ALDH1L1 expression is an independent predictor of overall survival in hepatocellular carcinoma patients [27]. A recent study in intrahepatic cholangiocellular carcinoma showed that low ALDH1A1 expression was linked to worse survival, an increased risk of death, and an unfavorable impact on overall survival [30]. In a study by Costa et al., a gradual loss of ALDH1A1 expression was reported in OSCC patients compared to oral leukoplakia patients, suggesting that lower levels of ALDH1A1 may predict carcinogenesis in oral lesions [28]. Our results indicate that low ALDH1A1 expression correlates with decreased disease-specific survival. The discrepancy between ALDH1A1 expression in OSCC and survival outcomes across various studies suggests a dual role for this biomarker, highlighting the need for further investigation and the potential development of new diagnostic and therapeutic strategies to manage devastating conditions like TSCC.

Lymph node metastasis has been discussed as a key prognostic factor in OSCC patients [31]. Regional lymph node metastasis was associated with lower ALDH1A1 expression in our study, unlike distant metastasis. Rao et al. demonstrated a significant correlation between lymph node metastasis and ALDH1 expression in OSCC [32]. Other studies also confirmed the relation between high ALDH1 expression and lymph node metastasis in OSCC [33,34]. These results are inconsistent with our findings, which could relate to the assessment of a subgroup of ALDH1, specifically ALDH1A1. Conversely, Gupta et al. found that high ALDH1A1 expression correlated with higher nodal stages [13]. Xu et al. suggested that ALDH1A1 is a potential biomarker for predicting lymph node metastasis in HNSCC patients [26]. ALDH1A1 expression and its association with lymph node metastasis have not been previously assessed in TSCC, which may explain the inconsistencies.

The discrepancy among the DOI, tumor thickness, and T category as prognostic factors for OSCC has gained significant attention in recent years [35,36]. A meta-analysis conducted by Caldeira et al. revealed that DOI can prognosticate N, recurrence, and chance of survival [14]. Tsai et al. concluded that DOI is a crucial prognostic factor in stage I OSCC, unlike tumor size [37]. Our study exhibited no relationship between DOI and OS, DSS, or RFS. There is no widely accepted cut-off value for DOI as a prognostic factor in TSCC. We performed Cox regression analysis on two DOI categories: one based on the WHO classification with a 10mm cut-off, and another based on the Tam et al. study, suggesting an 8mm cut-off for TSCC [16]. Incorrect measurement methods of DOI may affect the relationship between DOI and prognosis [31]. Lee et al. reported an association between DSS and the T category of the 8th edition of the AJCC [36], consistent with our results, highlighting the relationship between DSS and the T category.

WPOI, as a trendy prognostic marker, is associated with a worse prognosis in OSCC [38,39]. Elseragy et al. showed that WPOI has a prognostic value in early TSCC [40]. Dolens et al. conducted a systematic review and meta-analysis of some histopathological features, including WPOI, impacting OSCC prognosis [41]. They reported poor OS, DSS, and DFS concerning WPOI4 and WPOI5. Unlike the literature discussed above, our study found no relationship between WPOI and ALDH1A1 expression or survival rates.

There are some limitations and additional suggestions for our study. IHC evaluated ALDH1A1 in our research, but it is less sensitive than RNA-based assessment for quantifying protein levels. Isolating RNA for ALDH1A1 assessment may affect the results. In a more comprehensive study, all oral cavity sites, including the tongue, should be examined for ALDH1A1 expression, as different prognoses are linked to these sites. Our study included only TSCC cases. Serum levels of ALDH1A1 in patients should be compared with tissue levels in future studies. Additionally, comparing ALDH1A1 levels between a control group and dysplastic oral tumors is necessary.

## Conclusion

Reduced ALDH1A1 expression in TSCC has been associated with regional lymph node metastasis and decreased disease-specific survival. Our results diverge from the majority of prior studies examining ALDH1A1 expression in OSCC, which demonstrated a correlation between elevated ALDH1A1 levels and poorer prognoses in head and neck cancers. Low ALDH1A1 expression may indicate more aggressive disease, but the precise role of ALDH1A1 in TSCC warrants further investigation in larger populations, with comparisons to other CSC markers and an emphasis on distinct OSCC sublocations, as ALDH1A1 expression may vary across OSCC subtypes.

## Supporting information

S1 Table Anonymized dataset. De-identified data set to replicate study findings.
(DOCX)
