# Supplementary material for: Low ALDH1A1 expression in relation to nodal metastasis and survival in tongue squamous cell carcinoma
Source: PLoS One. 2026 Apr 10;21(4):e0345274. doi: 10.1371/journal.pone.0345274 (PMC13068279; doi:10.1371/journal.pone.0345274)
Supplement: S1 Table — De-identified data set to replicate study findings. (DOCX) [file pone.0345274.s001.docx]

| **Patient** | **Topography** | **Age (years)** | **Sex** | **Recurrence** | **Death** | **Recurrence-free survival (months)** | **Time to recurrence (months)** | **Time to death (months)** | **Reason of death** | **Inflammation** | **Grade** | **DOI (mm)** | **WPOI** | **Color intensity** | **Color percentage** | **ALDH1A1 expression** | **PNI** | **Stage** | **T** | **N** | **M** |
| --- | --- | --- | --- | --- | --- | --- | --- | --- | --- | --- | --- | --- | --- | --- | --- | --- | --- | --- | --- | --- | --- |
| 1 | C02.9 | 60 | Male | No | Yes | 16 |  | 16 | Disease-related | 3 | 2 | 13 | 5 | 3 | 2 | High | Present | 4 | 4 | 2 | 0 |
| 2 | C02.9 | 74 | Female | No | Yes | 12 |  | 12 | Disease-related | 2 | 2 | 6 | 2 | 1 | 2 | low | Absent | 2 | 2 | 0 | 0 |
| 3 | C02.9 | 39 | Male | No | Yes | 26 |  | 26 | Disease-related | 3 | 1 | 5 | 4 | 2 | 3 | High | Present | 4 | 4 | 1 | 0 |
| 4 | C02.2 | 57 | Female | No | No | 53 |  |  |  | 3 | 2 | 7 | 3 | 2 | 1 | low | Present | 2 | 2 | 0 | 0 |
| 5 | C02.1 | 66 | Female | No | No | 54 |  |  |  | 3 | 2 | 16 | 4 | 3 | 3 | High | Present | 3 | 2 | 1 | 0 |
| 6 | C02.9 | 74 | Male | No | No | 58 |  |  |  | 3 | 2 | 8 | 3 | 1 | 2 | low | Absent | 3 | 2 | 1 | 0 |
| 7 | C02.1 | 55 | Female | No | No | 57 |  |  |  | 3 | 3 | 4 | 4 | 2 | 1 | low | Present | 3 | 1 | 1 | 0 |
| 8 | C02.9 | 73 | Female | No | Yes | 16 |  | 16 | Disease-related | 2 | 1 | 7 | 2 | 1 | 1 | low | Absent | 3 | 1 | 1 | 0 |
| 9 | C02.9 | 80 | Female | No | Yes | 12 |  | 12 | Disease-related | 2 | 2 | 11 | 3 | 2 | 3 | High | Present | 3 | 1 | 0 | 0 |
| 10 | C02.9 | 42 | Female | No | Yes | 13 |  | 13 | Disease-related | 1 | 3 | 7 | 4 | 1 | 1 | low | Present | 3 | 2 | 1 | 0 |
| 11 | C02.9 | 50 | Male | Yes | Yes | 6 | 6 | 11 | Disease-related | 2 | 2 | 9 | 5 | 2 | 1 | low | Present | 2 | 2 | 0 | 0 |
| 12 | C02.1 | 58 | Male | No | Yes | 11 |  | 11 | Disease-related | 3 | 3 | 25 | 5 | 1 | 2 | low | Present | 4 | 4 | 3 | 1 |
| 13 | C02.9 | 60 | Male | Yes | No | 22 | 22 |  |  | 3 | 2 | 15 | 3 | 1 | 1 | low | Present | 3 | 2 | 1 | 0 |
| 14 | C02.9 | 43 | Female | Yes | Yes | 20 | 20 | 21 | Disease-related | 3 | 2 | 7 | 4 | 2 | 2 | High | Present | 3 | 3 | 1 | 0 |
| 15 | C02.1 | 72 | Female | Yes | Yes | 17 | 17 | 19 | Disease-related | 3 | 1 | 6 | 4 | 2 | 1 | low | Absent | 3 | 2 | 1 | 0 |
| 16 | C02.9 | 32 | Female | Yes | Yes | 8 | 8 | 15 | Disease-related | 3 | 1 | 11 | 4 | 2 | 1 | low | Present | 4 | 3 | 3 | 1 |
| 17 | C02.1 | 61 | Male | No | No | 42 |  |  |  | 2 | 1 | 5 | 4 | 1 | 2 | low | Absent | 3 | 2 | 1 | 0 |
| 18 | C02.0 | 82 | Male | No | Yes | 24 |  | 24 | Disease-related | 3 | 1 | 20 | 5 | 1 | 2 | low | Present | 3 | 3 | 0 | 0 |
| 19 | C02.1 | 92 | Female | No | Yes | 1 |  | 1 | Disease-related | 3 | 2 | 6 | 3 | 1 | 2 | low | Absent | 3 | 1 | 1 | 0 |
| 20 | C02 | 65 | Female | No | No | 45 |  |  |  | 3 | 3 | 10 | 4 | 2 | 3 | High | Present | 3 | 2 | 1 | 0 |
| 21 | C02.1 | 79 | Male | No | No | 49 |  |  |  | 3 | 3 | 9 | 4 | 1 | 2 | low | Present | 4 | 3 | 2 | 0 |
| 22 | C02.9 | 83 | Female | No | Yes | 3 |  | 3 | Disease-related | 3 | 2 | 18 | 5 | 1 | 1 | low | Present | 4 | 3 | 2 | 0 |
| 23 | C02.1 | 76 | Male | No | Yes | 12 |  | 12 | Disease-related | 3 | 1 | 7 | 3 | 1 | 3 | High | Present | 4 | 3 | 2 | 0 |
| 24 | C02.3 | 60 | Female | Yes | Yes | 5 | 5 | 8 | Disease-related | 3 | 3 | 11 | 4 | 1 | 1 | low | Present | 4 | 3 | 2 | 0 |
| 25 | C02.9 | 73 | Female | No | Yes | 8 |  | 8 | Disease-related | 3 | 3 | 5 | 4 | 0 | 0 | low | Present | 3 | 1 | 1 | 0 |
| 26 | C02.1 | 84 | Male | No | Yes | 2 |  | 2 | Disease-related | 3 | 2 | 18 | 3 | 1 | 2 | low | Present | 3 | 3 | 1 | 0 |
| 27 | C02.9 | 71 | Female | No | No | 43 |  |  |  | 3 | 2 | 10 | 3 | 1 | 1 | low | Absent | 3 | 2 | 1 | 0 |
| 28 | C02.1 | 51 | Female | No | No | 41 |  |  |  | 3 | 3 | 2 | 5 | 1 | 2 | low | Absent | 1 | 1 | 0 | 0 |
| 29 | C02.9 | 50 | Female | Yes | Yes | 13 | 13 | 15 | Disease-related | 3 | 3 | 5 | 4 | 3 | 3 | High | Present | 4 | 2 | 1 | 1 |
| 30 | C02.9 | 61 | Female | No | No | 50 |  |  |  | 2 | 2 | 11 | 4 | 3 | 2 | High | Present | 3 | 3 | 0 | 0 |
| 31 | C02.2 | 50 | Male | No | No | 49 |  |  |  | 3 | 2. | 7 | 4 | 3 | 3 | High | Absent | 2 | 2 | 0 | 0 |
| 32 | C02.9 | 43 | Female | No | No | 41 |  |  |  | 2 | 3 | 6 | 5 | 2 | 1 | low | Present | 4 | 3 | 2 | 0 |
| 33 | C02.1 | 39 | Male | No | No | 41 |  |  |  | 3 | 1 | 9 | 4 | 2 | 3 | High | Present | 3 | 2 | 1 | 0 |
| 34 | C02 | 78 | Female | Yes | Yes | 17 | 17 | 21 | Disease-related | 3 | 1 | 10 | 4 | 3 | 0 | low | Absent | 4 | 1 | 1 | 1 |
| 35 | C02.1 | 37 | Male | Yes | Yes | 11 | 11 | 18 | Disease-unrelated | 3 | 1 | 8 | 4 | 3 | 2 | High | Absent | 2 | 2 | 0 | 0 |
| 36 | C02.2 | 28 | Male | No | No | 35 |  |  |  | 3 | 1 | 11 | 4 | 2 | 1 | low | Absent | 4 | 3 | 2 | 0 |
| 37 | C02.9 | 48 | Female | No | No | 38 |  |  |  | 2 | 2 | 5 | 5 | 1 | 2 | low | Present | 1 | 1 | 0 | 0 |
| 38 | C02.9 | 59 | Female | No | No | 40 |  |  |  | 3 | 3 | 3 | 2 | 0 | 0 | low | Absent | 2 | 2 | 0 | 0 |
| 39 | C02.9 | 62 | Female | No | No | 34 |  |  |  | 2 | 2 | 6 | 4 | 2 | 2 | High | Absent | 2 | 2 | 0 | 0 |
| 40 | C02.1 | 39 | Male | No | No | 33 |  |  |  | 3 | 1 | 4 | 3 | 2 | 2 | High | Absent | 1 | 1 | 0 | 0 |
| 41 | C02.1 | 55 | Female | No | No | 35 |  |  |  | 3 | 2 | 4 | 5 | 2 | 2 | High | Absent | 1 | 1 | 0 | 0 |
| 42 | C02.9 | 56 | Male | No | No | 36 |  |  |  | 3 | 3 | 10 | 4 | 2 | 2 | High | Present | 3 | 3 | 0 | 0 |
| 43 | C02.1 | 29 | Male | Yes | No | 23 | 23 |  |  | 2 | 1 | 4 | 3 | 1 | 1 | low | Present | 3 | 2 | 1 | 0 |
| 44 | C02.1 | 45 | Male | No | No | 39 |  |  |  | 3 | 1 | 5 | 3 | 2 | 2 | High | Absent | 3 | 2 | 1 | 0 |
| 45 | C02.1 | 24 | Male | No | No | 38 |  |  |  | 3 | 2 | 6 | 4 | 3 | 1 | High | Absent | 2 | 2 | 0 | 0 |
| 46 | C02.3 | 38 | Male | Yes | No | 8 | 8 |  |  | 3 | 2 | 7 | 4 | 3 | 3 | High | Absent | 2 | 2 | 0 | 0 |
| 47 | C02.0 | 24 | Female | No | No | 62 |  |  |  | 3 | 2 | 4 | 4 | 3 | 3 | High | Present | 1 | 1 | 0 | 0 |
| 48 | C02.9 | 101 | Male | No | Yes | 44 |  | 44 | Disease-unrelated | 2 | 1 | 10 | 4 | 3 | 2 | High | Present | 4 | 2 | 3 | 0 |
| 49 | C02.9 | 60 | Male | Yes | No | 35 | 35 |  |  | 2 | 2 | 7 | 4 | 3 | 3 | High | Present | 1 | 1 | 0 | 0 |
| 50 | C02.9 | 63 | Male | Yes | No | 26 | 26 |  |  | 2 | 3 | 12 | 5 | 2 | 3 | High | Present | 3 | 2 | 0 | 0 |
| 51 | C02 | 48 | Female | No | No | 56 |  |  |  | 1 | 1 | 6 | 4 | 3 | 3 | High | Absent | 2 | 1 | 0 | 0 |
| 52 | C02.1 | 42 | Male | No | No | 55 |  |  |  | 3 | 1 | 8 | 5 | 3 | 3 | High | Absent | 2 | 2 | 0 | 0 |
| 53 | C02.1 | 59 | Female | No | No | 56 |  |  |  | 3 | 2 | 11 | 4 | 2 | 3 | High | Absent | 3 | 2 | 0 | 0 |
| 54 | C02.9 | 68 | Male | No | Yes | 36 |  | 36 | Disease-unrelated | 2 | 1 | 10 | 5 | 3 | 3 | High | Absent | 3 | 2 | 0 | 0 |
| 55 | C02.0 | 30.00 | Female | No | No | 56 |  |  |  | 2 | 1 | 15 | 5 | 2 | 1 | low | Present | 3 | 2 | 0 | 0 |
